# Statement of Retraction: Monkeypox virus induces ferroptosis to facilitate viral replication and promotes inflammatory responses

**DOI:** 10.1080/22221751.2026.2710009

**Published:** 2026-08-06

We, the Editors and Publisher of *Emerging Microbes & Infections* have retracted the following article:

Chuai, X., Wang, Y., Wang, C., Ye, T., Shen, X., Zhou, J., Zhang, K., Zhao, B., Wu, Y., Li, W., Cai, Z. and Chiu, S. (2025). Monkeypox virus induces ferroptosis to facilitate viral replication and promotes inflammatory responses. *Emerging Microbes & Infections*, *14*(1). https://doi.org/10.1080/22221751.2025.2522877

Following publication, the authors contacted the editorial office with a request to correct Figure S1H as a duplication of Figure 1J.

Further investigation by the publisher and journal has identified concerns regarding the RNA methodology and the Western Blot results. When approached for an explanation, the authors responded, however concerns remain.

As verifying the validity of published work is core to the integrity of the scholarly record, we are therefore, retracting the article. The authors do not agree with the retraction.

We have been informed in our decision-making by our editorial policies and the COPE guidelines.

The retracted articles will remain online to maintain the scholarly record, but they will be digitally watermarked on each page as ‘Retracted’.

